# Activin A Inhibits MPTP and LPS-Induced Increases in Inflammatory Cell Populations and Loss of Dopamine Neurons in the Mouse Midbrain *In Vivo*

**DOI:** 10.1371/journal.pone.0167211

**Published:** 2017-01-25

**Authors:** Sandy Stayte, Peggy Rentsch, Anna R. Tröscher, Maximilian Bamberger, Kong M. Li, Bryce Vissel

**Affiliations:** 1 Neuroscience Department, Garvan Institute of Medical Research, Sydney, New South Wales, Australia; 2 FH Krems University of Applied Science, Krems, Austria; 3 Pharmacology Department, Bosch Institute, Sydney Medical School, The University of Sydney, Sydney, Australia; 4 School of Life Sciences, University of Technology Sydney, Sydney, Australia; Hudson Institute, AUSTRALIA

## Abstract

Parkinson’s disease is a chronic neurodegenerative disease characterized by a significant loss of dopaminergic neurons within the substantia nigra pars compacta region and a subsequent loss of dopamine within the striatum. A promising avenue of research has been the administration of growth factors to promote the survival of remaining midbrain neurons, although the mechanism by which they provide neuroprotection is not understood. Activin A, a member of the transforming growth factor β superfamily, has been shown to be a potent anti-inflammatory following acute brain injury and has been demonstrated to play a role in the neuroprotection of midbrain neurons against MPP^+^-induced degeneration *in vitro*. We hypothesized that activin A may offer similar anti-inflammatory and neuroprotective effects in *in vivo* mouse models of Parkinson’s disease. We found that activin A significantly attenuated the inflammatory response induced by both MPTP and intranigral administration of lipopolysaccharide in C57BL/6 mice. We found that administration of activin A promoted survival of dopaminergic and total neuron populations in the pars compacta region both 8 days and 8 weeks after MPTP-induced degeneration. Surprisingly, no corresponding protection of striatal dopamine levels was found. Furthermore, activin A failed to protect against loss of striatal dopamine transporter expression in the striatum, suggesting the neuroprotective action of activin A may be localized to the substantia nigra. Together, these results provide the first evidence that activin A exerts potent neuroprotection and anti-inflammatory effects in the MPTP and lipopolysaccharide mouse models of Parkinson’s disease.

## Introduction

Parkinson’s disease (PD) is a chronic neurodegenerative disease characterized by significant loss of dopaminergic neurons within the substantia nigra pars compacta (SNpc) and a subsequent loss of dopamine (DA) within the striatum. While the use of levodopa remains the gold standard in the treatment of the motor symptoms, this therapeutic strategy does not address the underlying nigral degeneration. Considerable research has therefore been directed at aiming to identify therapeutic treatments that are able to overcome this issue. One of the most promising avenues in recent years has been the administration of growth factors to promote the survival of remaining midbrain neurons, however the mechanism by which they provide neuroprotection has yet to be fully understood.

Growth factors have long been known to be critical in the induction, specification, survival and maturation of developing neurons within the CNS. Following injury, growth factors and their receptors have been shown to increase in concentration, suggesting they are part of an endogenous mechanism aimed at an attempted regenerative response [1]. While their exact mechanisms of action still remain to be fully elucidated, growth factors have been demonstrated to act as neuroprotective molecules against cytotoxic cell damage via upregulation of calcium buffering proteins, antioxidant enzymes, and anti-apoptotic factors [2].

Activin A is a member of the transforming growth factor (TGF)-β superfamily, which is known to be involved in development, repair of tissues and organs, and in neuroinflammation. In addition to its known neuroprotective effects in the hippocampus [3], activin A has been shown to exert neuroprotective effects in midbrain neurons *in vitro*, with dopaminergic neuronal cultures that have been treated with activin A maintaining a higher number of tyrosine hydroxylase (TH) positive neurons 8 days after culture compared to untreated controls, representing a 2.5 fold increase in the survival of dopaminergic neurons [4]. Furthermore, when administered prior to exposure with the parkinsonian neurotoxin MPP^+^, activin A treatment resulted in an approximate 50% survival rate of dopaminergic neurons [4]. In addition, exogenous application and transfection with activin A significantly increased human SHSY5Y neuroblastoma cell viability following serum withdrawal and 6-OHDA administration, with this neuroprotection inhibited by the activin-binding protein follistatin [5].

More recently activin A was shown to provide significant neuroprotection of midbrain neuron populations *in vivo*, with exogenous activin A significantly attenuating degeneration induced by unilateral 6-OHDA lesioning [6]. While these results suggest that activin A may also protect against other parkinsonian toxins *in vivo*, the mechanism by which this growth factor exerts its neuroprotection remains to be found.

Neuroinflammation has steadily been gaining prominence as a significant factor in the pathogenesis of PD [7–11]. While there have been studies demonstrating the effect of growth factors on the inflammatory response in both the healthy and PD brain [12–15], a study conducted by Abdipranoto-Cowley et al., (2009) provided the first evidence for a potent anti-inflammatory effect of activin A in the brain after acute degeneration. Following intracerebroventricular (i.c.v) injection of kainic acid and a subsequent inflammatory response, the administration of exogenous activin A was able to exert its anti-inflammatory effects in the CNS by suppressing microglial numbers, microglial activation, pro-inflammatory cytokine release, and gliosis both *in vivo* and *in vitro* [16]. This centrally derived anti-inflammatory process was significantly attenuated by the administration of follistatin, with animals receiving follistatin displaying increased microglial numbers and gliosis and at levels significantly higher than those induced by kainic acid alone. Furthermore, activin A was able to profoundly limit the response induced by the direct inflammatory stimulator lipopolysaccharide (LPS) [16]. This study is the first to provide evidence that exogenous application of activin A prior to 1-methyl-4-phenyl-1,2,3,6-tetrahydropyridine (MPTP) administration provides significant protection of midbrain neurons from degeneration *in vivo* and furthermore provides evidence that its neuroprotective actions may derive from its potent anti-inflammatory properties.

## Materials and Methods

### Animals

C57BL/6 male mice aged 11 weeks were obtained from Australian BioResources (Moss Vale, Australia). Mice were housed at a maximum five mice per cage for 1 week, until the study began, at which time mice were housed individually. Mice were kept on a 12-hour light/dark cycle and access to food and water *ad libitum*. All animal procedures were performed with the approval of the Garvan Institute and St. Vincent’s Hospital Animal Ethics Committee under approval numbers 09/14 and 12/36, in accordance with the Australian National Health and Medical Research Council animal experimentation guideline and the local Code of Practice for the Care and Use of Animals for Scientific Purposes (2004). All surgery was performed under ketamine/xylazil anesthesia, and all efforts were made to minimize suffering.

### MPTP administration

Animals received subcutaneous injections of 20 mg/kg of MPTP (Sigma Aldrich) as described in detail previously [6]. All mice were sacrificed either 8 days or 8 weeks after the final administration of MPTP or saline.

### Intranigral injection of lipopolysaccharide

Mice were anaesthetized with a mixture of ketamine (8.7 mg/ml; Mavlab) and xylazil (2 mg/ml; Troy Laboratories Pty Ltd) and placed in a stereotaxic apparatus (Kopf Instruments). Mice were then injected with 2 μl of 5 μg/μl of LPS, or vehicle control, at a rate of 0.5 μl/minute in the substantia nigra at the following coordinates relative to bregma: AP -3.0, ML +1.3, DV -4.7. All mice were sacrificed 15 days after lesioning.

### Osmotic micropump implantation

The day prior to MPTP, or the day after LPS administration, animals were anaesthetized and placed in a stereotaxic apparatus as described above. For all MPTP experiments, osmotic micro-pumps (Model 1007D; Alzet) were filled with 24.5 ng/μl (295 ng total daily dose) of recombinant human/mouse/rat activin A (R&D Systems) or vehicle control (1x PBS) and implanted subcutaneously along the back of the neck. For all LPS experiments osmotic micro-pumps (Model 1002D; Alzet) were filled with 49 ng/μl (295 ng total daily dose, corresponding to dose used in MPTP experiments) of activin A or vehicle control. An infusion cannula (PlasticsOne) connected to the micropump was placed in the right lateral ventricle at AP -0.26, ML +1.0, DV -2.8 relative to bregma. For all long-term MPTP studies the pump was removed 1 week later and the cannula tubing sealed with heat.

### Immunohistochemistry

Mice were anaesthetized and transcardially perfused with ice-cold phosphate-buffered saline (PBS) and 4% paraformaldehyde. Brains were harvested and processed as described in detail previously [6]. Sections were incubated in the following primary antibodies: monoclonal mouse tyrosine hydroxylase (TH, 1:1000 Sigma Aldrich cat # T2928), monoclonal mouse neuronal nuclei (NeuN 1:500, Merck Millipore, cat # MAB377), polyclonal rabbit glial fibrillary acidic protein (GFAP 1:300, Dako cat # Z033401), polyclonal rabbit Iba1 (1:1000, Novachem, cat # 019–19741) for 72 hours at 4°C. All sections were then incubated in the respective biotin-labeled secondary antibodies (1:250, Sapphire Bioscience cat # AB6813, Life Technologies, cat # B-2770) overnight at 4°C followed by incubation in avidin-biotin complex (Vector Laboratories) at room temperature for 1 hour. TH immunolabeling was detected with 3,3’-Diaminobenzidine (DAB, Abacus) until desired staining achieved. NeuN, GFAP and Iba1 immunolabeling was detected with DAB intensified with nickel ammonium sulfate and counterstained with polyclonal rabbit anti-TH (1:1000, Merck Millipore cat # AB152) that was detected with Nova-Red (Abacus) to outline the substantia nigra region.

### Stereology

Quantification of SNpc cell population estimates was performed using the optical fractionator method and the use of Stereo Investigator 7 software (MBF Bioscience). For the estimations of TH positive populations a counting frame of 60 μm x 60 μm and a grid size of 113 μm x 73 μm was used. For the estimations of NeuN positive populations a counting frame of 65 μm x 65 μm and a grid size of 155 μm and 125 μm was used. For the estimation of GFAP and Iba1 positive populations a counting frame of 58 μm x 58 μm and a grid size of 61 μm x 61 μm was used. For all cell types the guard zone height used was 5 μm and dissector height used was 10 μm with every third section sampled to a total of 10 sections for TH and NeuN quantification, and every sixth section sampled to a total of 5 sections for GFAP and Iba1 quantification. Coefficient of error attributable to the sampling was calculated according to Gundersen and Jensen [17]. Errors ≤0.10 were regarded as acceptable. The SNpc was delineated from -2.8 to -3.88 mm relative to bregma based on the Paxinos atlas for the mouse brain [18].

### Catecholamine analysis

Animals were sacrificed by cervical dislocation either 8 days or 8 weeks after the administration of MPTP or saline, the brains removed and the striatum rapidly dissected out and snap frozen. Striata were analysed for norepinephrine (NE), dopamine (DA), 3,4-dihydroxyphenylacetic acid (DOPAC), and homovanillic acid (HVA) via HPLC as described previously [6]. Catecholamine levels were standardized to protein levels measured via Bradford assay and recorded as ng/μg of protein. All standards for the Bradford assay were prepared in freshly made 0.2 M PCA + 0.1% L-cysteine.

### Western blotting

Animals were sacrificed by cervical dislocation either 8 days or 8 weeks after the administration of MPTP or saline or 15 days after the administration of LPS, the brains removed and the striatum rapidly dissected out and snap frozen. Striata were sonicated in protease inhibitor cocktail (Sigma) diluted at a concentration of 1:1000 in RIPA buffer (Sigma) and centrifuged at 16,000g for 15 minutes at 4°C. For all samples, 10 μg was loaded onto 4–12% Bis-Tris gels (Life Technologies) and protein was separated by running the gel at 180V for approximately 45 minutes. Following transfer to PDVF membranes (Life Technologies), membranes were blocked in 10% skim milk solution for 1 hour at room temperature and then incubated in 0.1% BSA solution containing either polyclonal rabbit phosphorylated TH(Ser40) (Thermo Fischer Scientific cat # sc-135715), polyclonal rabbit phosphorylated TH(Ser31) (Thermo Fischer Scientific cat # sc-135714) or monoclonal rat dopamine transporter (DAT) antibody (Merck Millipore cat # MAB369) at a concentration of 1:1000 and kept at 4°C overnight on an orbital shaker. Following washing in buffer containing 1 x TBS buffer without triton + 0.1% Tween 20 for 1 hour, membranes were incubated in 5% skim milk solution containing the respective HRP secondary antibodies (1:1000, Sapphire Bioscience cat # AB97057 and Merck Millipore cat # AP132P) for 1 hour at room temperature. Membranes were then visualized using Novex ECL Chemiluminescent Substrate Reagent Kit (Life Technologies) and exposed to x-ray film (FUJIFILM). Films were scanned and analyzed using Image J Software.

All membranes probed for p-TH(Ser40) and p-TH(Ser31) were then stripped and re-probed with monoclonal mouse anti-TH (1:1000 Sigma Aldrich, cat # T2928) followed by stripping and re-probing with monoclonal rabbit anti-GAPDH (1:1000, Cell Signaling cat # 2118). All membranes probed for DAT were stripped and re-probed for anti-GAPDH. Films were scanned and analyzed using Image J Software. For quantification of all phosphorylation western blots, the raw values were first expressed as a ratio to total TH, standardized to GAPDH and then adjusted with the values obtained from PBS + Saline treated mice. For quantification of DAT western blots, raw values were standardized to GAPDH and adjusted with the values obtained from PBS + Saline treated mice.

### Activin ELISA

Mice were sacrificed by cervical dislocation and the striatum and midbrain (substantia nigra + ventral tegmental area) rapidly dissected out 24 hours after the last MPTP (or saline) injection. Tissues were homogenized in lysis buffer containing 5 mM Tris-HCL, pH 8.0, 0.32 M sucrose, and protease inhibitor cocktail (Sigma Aldrich, Australia), and homogenates were centrifuged at 17,000xg at 4°C for 10 minutes. Supernatant was collected and assayed for quantification of total protein via Bradford assay. Activin A levels were assayed by ELISA kit (Quantikine Activin A assay, R&D systems) according to manufacturer’s instructions.

### Statistical analysis

All statistical analysis was performed using GraphPad Prism Version 6.0 (GraphPad Software, Inc). Differences between means was assessed, as appropriate, by two- or one-way ANOVA followed by Bonferroni *post hoc* analysis, or via unpaired t test or Mann-Whitney testing.

## Results

### Activin A increases survival of dopaminergic neurons in the substantia nigra following MPTP

**Fig 1:**
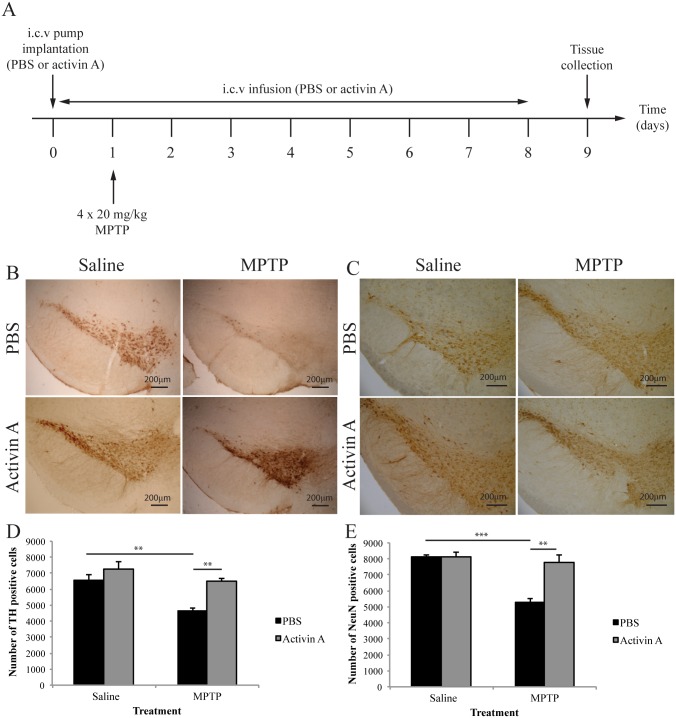
Activin A protects nigral neurons against MPTP-induced cell death. (A) Experimental timeline. (B) Representative images of TH-immunoreactive neurons in the substantia nigra pars compacta (SNpc). (C) Representative images of NeuN-immunoreactive neurons in the SNpc. (D) Stereological quantification of TH-immunoreactive neurons in SNpc demonstrates activin A protects dopaminergic neurons against MPTP toxicity. (E) Stereological quantification of NeuN-immunoreactive neurons in the SNpc demonstrates activin A protects total neuron numbers against MPTP induced toxicity. All values represent the mean ± SEM. *p<0.05, **p<0.01, ***p<0.001. Scale bar represents 200μm. N = 5/group.

It has been previously shown that activin A is able to protect against MPP^+^-induced degeneration *in vitro* [4]. To investigate if exogenous application of activin A exerts the same neuroprotective effects *in vivo*, surviving midbrain dopaminergic neurons, identified by TH immunoreactivity, were quantified in the SNpc region via stereology. As expected, MPTP administration resulted in a significant loss of TH positive neurons (Interaction F_(1,16)_ = 3.118 *p* = 0.0965; Toxin F_(1,16)_ = 16.62 *p*<0.001; Drug F_(1,16)_ = 15.66 *p*<0.01, n = 5/group) with animals receiving PBS displaying significantly fewer TH positive cells following MPTP compared to their saline controls (*p*<0.01). However, i.c.v infusion of activin A significantly increased the number of surviving TH positive neurons against MPTP induced toxicity (*p*<0.01), without altering baseline levels of dopaminergic cell numbers in saline treated animals (*p* = 0.2816 Fig 1B and 1D). Furthermore, the number of DA neurons in animals receiving activin A and MPTP was not statistically different from those receiving activin A and saline (*p* = 0.2434), suggesting a potent neuroprotective effect of activin A.

### Activin A increases survival of nigral neuron populations following MPTP

It has been demonstrated that expression of TH may decrease during times of cell stress that occurs during pathological events induced by Parkinsonian toxins such as MPTP [19–21]. It is therefore necessary to control for the loss of this phenotypic marker in the absence of cell death. To ensure that the neuroprotective effect of activin A in dopaminergic neurons against MPTP-induced degeneration was not simply a result of decreased TH expression in vehicle treated animals, we also quantified the number of NeuN positive cells in the SNpc. Two-way ANOVA revealed a significant interaction between toxin and drug treatment (F_(1,16)_ = 15.21 *p*<0.01, n = 5/group), suggesting an effect of activin A on MPTP-induced cell loss. Therefore separate t-tests of toxin and drug treatment were conducted. Administration of MPTP significantly decreased the number of positively stained NeuN cells in animals receiving vehicle (t = 12.48, df = 8, *p*<0.0001 Fig 1C and 1D), however activin A significantly protected against this loss (t = 4.656, df = 8, *p*<0.01) without altering the baseline number of NeuN positive cells in saline treated animals (t = 0.1176, df = 8, *p* = 0.9093). Furthermore the number of NeuN positive cells did not differ in animals receiving activin A and lesioned with MPTP, compared to their saline controls (t = 0.6578, df = 8, *p* = 0.5291, Fig 1C and 1E). Combined, these results indicate a complete protection of total neuron numbers with activin A.

### Activin A does not alter striatal dopamine levels following MPTP

The loss of dopaminergic neurons within the SN in PD results in a subsequent loss of DA in the striatum. As we demonstrated that activin A treatment is able to protect against both dopaminergic and total neuron loss induced by MPTP toxicity, we hypothesized that activin A treatment would also result in a concurrent protection of striatal DA levels. We therefore quantified catecholamine levels from striatal tissue and analyses for DA, DOPA, HVA, and NE via HPLC coupled to an electrochemical detector was conducted.

**Fig 2:**
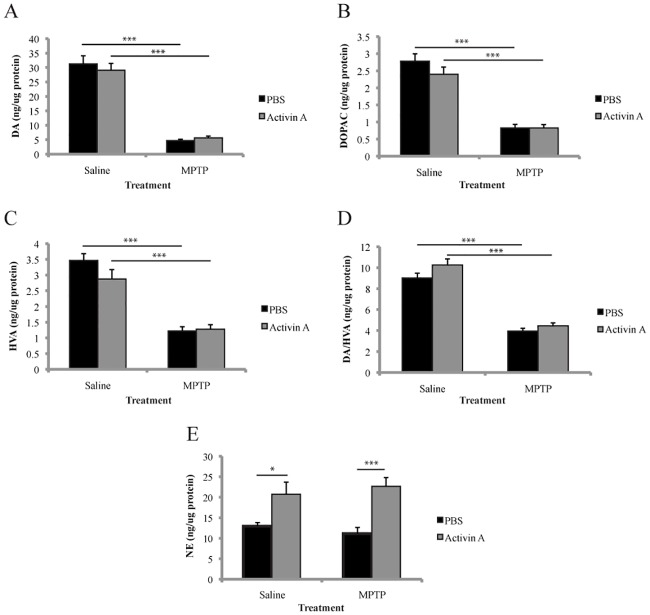
Activin A does not alter striatal dopamine levels following MPTP. HPLC-ECD quantification of catecholamine levels revealed that activin A does not alter striatal DA (A), DOPAC (B), or HVA (C) levels following MPTP administration. Activin A did not alter the catabolism of DA to HVA (D) following MPTP administration. Activin A significantly increased striatal NE levels (E). All values represent the mean ± SEM. *p<0.05, ***p<0.001. N = 5-7/group.

It has been well documented that MPTP-induced toxicity results in a significant decrease in striatal DA levels, an effect that we have replicated here (Fig 2A; F_(1,18)_ = 210.9 *p*<0.001, n = 5-7/group) with animals receiving PBS (*p*<0.001) and activin A (*p*<0.001) both displaying significant loss of striatal DA following MPTP. However, two-way ANOVA did not reveal any significant main effect of drug treatment on DA levels (F_(1,18)_ = 0.145 *p* = 0.7078) with no significant difference found in DA levels between MPTP treated animals receiving PBS and those receiving activin A (*p*>0.9999), suggesting that activin A does not protect against loss of DA levels following MPTP. Furthermore, this was not due to insufficient levels of activin A as ELISA analysis demonstrated that i.c.v delivery of activin A resulted in significant quantities of activin A in both the midbrain and striatum (S1 Fig).

Following its release from presynaptic terminals, DA either binds to and activates the DA receptors D1 to D5 [22], or is actively translocated from the extracellular space into presynaptic neurons and surrounding glial cells via the dopamine transporter where it is then either repackaged into synaptic vesicles or degraded into its metabolites DOPAC and HVA [23]. We therefore quantified the amount of these metabolites within the striatum in order to investigate if they were altered with the administration of activin A. Two-way ANOVA with *post hoc* Bonferroni corrections revealed no significant effect of activin A on baseline DOPAC (*p* = 0.2466) or HVA (*p* = 0.1877) levels (Fig 2B and 2C). While the administration of MPTP significantly decreased DOPAC levels (Fig 2B), there was no significant difference between MPTP-treated animals receiving PBS or activin A (*p*>0.9999). Similarly, MPTP administration resulted in a significant reduction in HVA levels (Fig 2C), however no significant difference was found between PBS and activin A groups treated with MPTP (*p*<0.9999). Furthermore, activin A does not alter the catabolism of DA to HVA, with two-way ANOVA analysis revealing no significant difference in the ratio of these two catecholamines between animals receiving PBS or activin A, treated with MPTP (Fig 2D *p* = 0.7630).

### Activin A increases striatal norepinephrine levels

Following conversion of DOPA to DA by DOPA decarboxylase, DA is transported into vesicles where it can also be converted to NE by dopamine β-hydroxylase. While not as extensively studied as DA, evidence suggests that NE may play a role in the degenerative process of PD [24,25]. We therefore also examined the levels of NE in the striatum following MPTP. Interestingly, two-way ANOVA revealed no significant effect of MPTP treatment on NE levels (F_(1,18)_ = 0.001491 *p* = 0.9696, n = 5-7/group), however a significant drug effect was found (F_(1,18)_ = 23.91 *p*<0.0001) suggesting a potential role of activin A on NE levels. *Post hoc* analysis with Bonferroni corrections demonstrated that activin A significantly increased levels of NE both in saline (*p*<0.05) and MPTP (*p*<0.001) treated groups (Fig 2E). However MPTP did not alter this increase in NE levels, with no significant difference between animals receiving activin A and treated with saline, and animals receiving activin A and treated with MPTP (*p*>0.9999).

### Tyrosine hydroxylase phosphorylation is altered by activin A following MPTP

Tyrosine hydroxylase can be regulated by phosphorylation at multiple serine residues, including serine 19, 31 and 40 by various kinases, resulting in increased stability and/or activity of the enzyme and subsequent increases in DA levels [26]. Our previous results demonstrated that while activin A is able to protect the DA producing cell bodies in the SN, there is no subsequent protection of DA levels in the striatum. To investigate if this failure to increase striatal DA levels resulted from alterations to tyrosine hydroxylase activity, we analyzed expression of ser40 and ser31 phosphorylated TH via immunoblotting.

**Fig 3:**
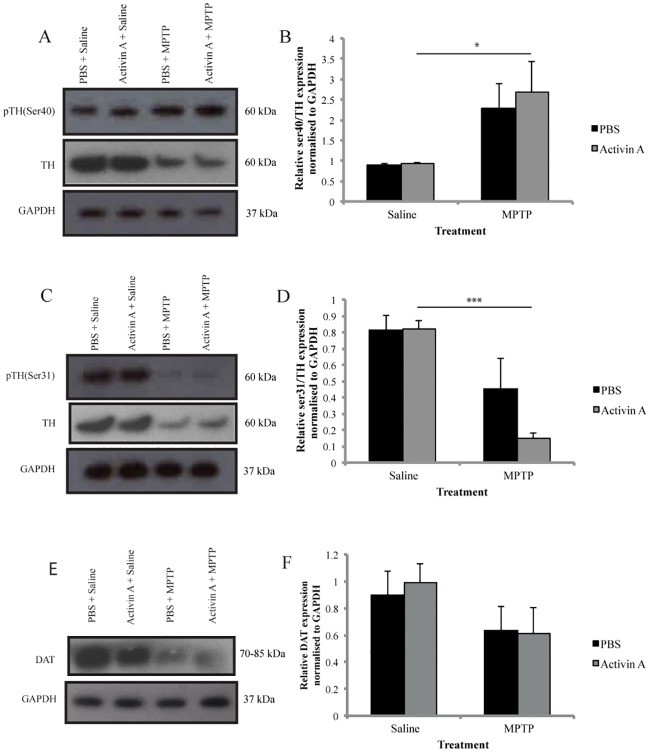
Activin A alters TH phosphorylation but not striatal DAT following MPTP. Western blot analysis of striatal protein extracts from animals receiving PBS or activin A 8 days after MPTP. Representative bands of TH, GAPDH and (A) p-TH(ser40) and (C) p-TH(ser31) expression. Quantification of p-TH(ser40)/TH (B) and p-TH(ser31)/TH (D) protein expression normalized to GAPDH (n = 6/group). (E) Representative bands of DAT and GAPDH expression. (F) Quantification of DAT protein expression normalized to GAPDH. All values represent the mean ± SEM. *p<0.05, ***p<0.001. N = 6-7/group.

Two-way ANOVA analysis revealed a significant overall effect of MPTP on both striatal p-TH(ser40) (F_(1,20)_ = 10.41 *p*<0.01, n = 6/group), and p-TH(ser31) expression (F_(1,20)_ = 21.74 *p*<0.001, n = 6/group). However no overall effect of drug treatment on either p-TH(ser40) (F_(1,20)_ = 0.2132 *p* = 0.6493) or p-TH(ser31) expression in the striatum (F_(1,20)_ = 1.745 *p* = 0.2014) was found. *Post hoc* analysis revealed no significant difference in serine phosphorylation in saline treated animals receiving either PBS or activin A (*p*>0.9999), indicating that activin A does not alter baseline levels of p-TH(ser40) or p-TH(ser31) expression (Fig 3A–3D). However, animals receiving activin A and MPTP displayed a significant increase in p-TH(ser40) expression compared to their saline treated controls (*p*<0.05 Fig 3B). In contrast, while MPTP did not alter p-TH(ser31) expression in animals receiving PBS (*p* = 0.0642), activin A significantly lowered expression levels compared to saline treated controls (*p*<0.001 Fig 3D). Together, these results indicate that activin A alters MPTP-induced changes in TH phosphorylation.

### Activin A does not protect striatal terminals against MPTP-induced degeneration

In the striatum, the DAT plays an important role for maintaining sufficient DA levels for release into the synaptic cleft, thus when striatal DAT loss reaches levels equivalent to those seen upon presentation of locomotor symptoms, a concomitant deficit in DA is produced [27–29]. To investigate if the inability of activin A to restore DA levels in the striatum following MPTP was due to a deficit in DAT, we quantified striatal DAT levels via immunoblotting.

Two-way ANOVA analysis, revealed no significant overall effect of MPTP (F_(1,21)_ = 3.999 *p* = 0.0586, n = 6-7/group), and no significant overall effect of drug treatment (F_(1,21)_ = 0.04795 *p* = 0.8288) on DAT expression. Furthermore, no significant difference was found in saline treated animals receiving either PBS or activin A (*p*>0.9999), indicating that activin A does not alter baseline levels of DAT expression (Fig 3E and 3F). These results suggest that unlike the neuroprotective effect seen in the SN, activin A does not protect the dopaminergic fibers in the striatum.

### Activin A protects dopamine neuron numbers 8 weeks following MPTP

Much like that seen in the early phases of human PD in which the dopaminergic system is able to compensate for the initial degeneration, numerous studies have demonstrated that spontaneous recovery is able to occur in rodents that have been rendered parkinsonian by MPTP [30–32]. To investigate if our effects of activin A are due to any potential neuroprotective properties or simply a result of spontaneous recovery in these animals, a second cohort of mice was quantified for TH positive cells in the SNpc 8 weeks after MPTP administration.

**Fig 4:**
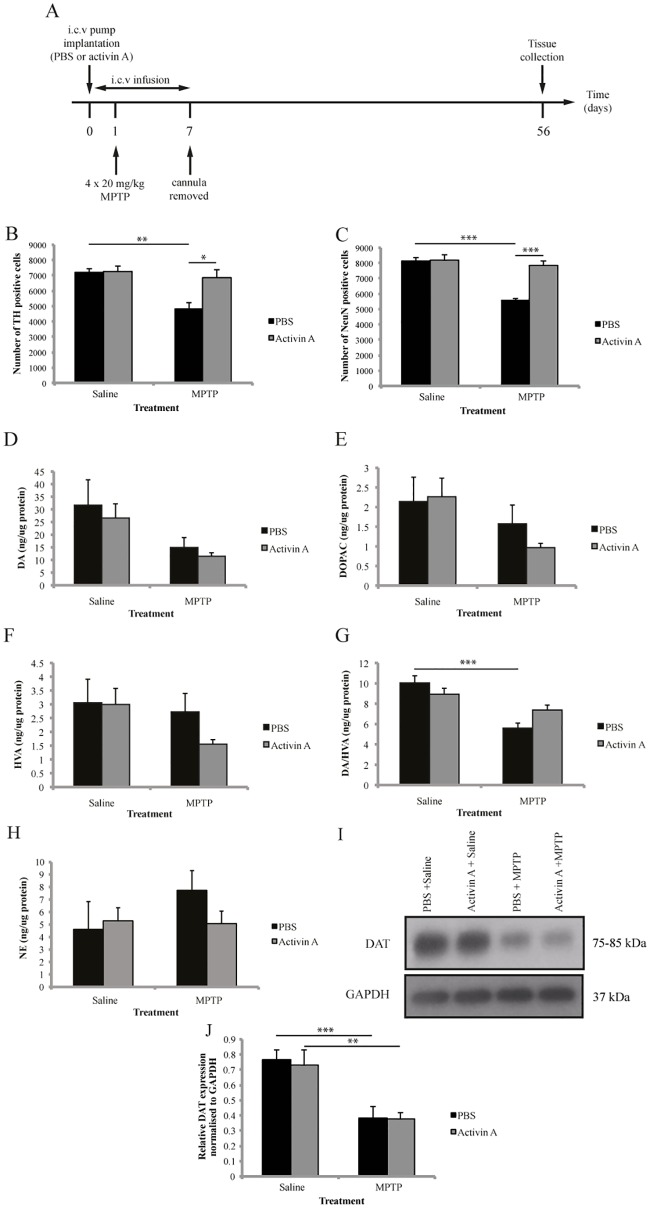
Activin A protects midbrain neurons up to 8 weeks post-MPTP. Stereological quantification demonstrates activin A protects SNpc dopaminergic neurons (A) and total neuron numbers (B) against long-term MPTP induced toxicity. No significant effect of activin A on striatal DA (C), DOPAC (D) or HVA levels (E) the turnover of DA to HVA (F) or striatal NE levels (G) was found 8 weeks post-MPTP. (H) Representative images of DAT and GAPDH expression. (I) Western blot analysis of striatal protein extracts revealed activin A does not protect DAT expression 8 weeks post-MPTP. All values represent the mean ± SEM. *p<0.05 **p<0.01, ***p<0.001. N = 3-13/group.

Two-way ANOVA revealed a significant interaction between toxin and drug treatment (F_(1,17)_ = 5.594 *p*<0.05, n = 5-6/group) suggesting a potential effect of activin A on the long-term toxicity of MPTP. Therefore, separate t-tests were conducted on toxin and drug treatment. As expected, activin A did not alter baseline levels of dopaminergic cells (t = 0.1115, df = 8, *p* = 0.9140), while MPTP significantly decreased the number of TH positive cells in animals receiving PBS (t = 4.747, df = 9, *p*<0.01 Fig 4B). Interestingly activin A significantly increased the number of surviving TH positive cells following MPTP (t = 3.000, df = 9 *p*<0.05) to a level that was not significantly different from their saline controls (t = 0.7139, df = 8, *p* = 0.4956) suggesting that short term administration of activin A is able to protect dopamine neurons up to 8 weeks following MPTP (Fig 4B).

### Activin A protects total neuron numbers 8 weeks following MPTP

We also examined the effect of activin A on total neuron numbers 8 weeks after MPTP administration by quantifying the number of NeuN positive cells within the SNpc. Two-way ANOVA revealed a significant interaction between toxin and drug treatment (F_(1,16)_ = 17.76 *p*<0.001, n = 5/group) suggesting a potential effect of activin A on the long-term survival of nigral neurons following MPTP. Therefore, separate t-tests were conducted on toxin and drug treatment. We demonstrated that, much like the results seen with TH, activin A did not alter baseline levels of neuron numbers at 8 weeks post MPTP (t = 0.2298, df = 8, *p* = 0.8240 Fig 4C). While MPTP significantly decreased the number of NeuN positive cells in animals receiving PBS (t = 8.720, df = 8, *p*<0.001), activin A significantly increased the survival of NeuN positive cells compared to controls (t = 7.127, df = 8, *p*<0.001 Fig 4C). Furthermore, there was no significant difference in NeuN positive cells in MPTP-treated animals receiving activin A compared to their saline controls (t = 0.9319, df = 8, *p* = 0.3786) indicating that short term administration of activin A is able to completely protect against MPTP-induced cell death up to 8 weeks after lesioning.

### Activin A does not alter striatal dopamine content 8 weeks following MPTP

To investigate if activin A alters striatal DA content past the initial degeneration phase, we analyzed the levels of striatal DA, DOPAC, and HVA 8 weeks after the last injection of MPTP. Two-way ANOVA analysis demonstrated a significant overall effect of MPTP on both DA (F_(1,13)_ = 4.732 *p*<0.05, n = 3-5/group) and DOPAC (F_(1,13)_ = 6.246 *p*<0.05, n = 3-5/group) levels, while no significant overall effect of MPTP was found on HVA levels (F_(1,13)_ = 1.535 *p* = 0.2373, n = 3-5/group). Furthermore, no significant overall effect of drug treatment on DA (F_(1,13)_ = 0.3358 *p* = 0.5722), DOPAC (F_(1,13)_ = 0.02681 *p* = 0.8725) or HVA (F_(1,13)_ = 0.7387 *p* = 0.4056) was found. However, *post hoc* analysis revealed no significant difference in DA or DOPAC levels between animals receiving PBS and activin A in both saline and MPTP-treated groups (Fig 4D–4F).

Interestingly, two-way ANOVA revealed a significant interaction between toxin and drug treatment in the ratio of DA to HVA (F_(1,13)_ = 5.323 *p*<0.05, n = 3-5/group) suggesting a potential effect of activin A on the catabolism of DA following MPTP. Therefore t-tests of toxin and drug were performed and it was found MPTP-treated animals receiving PBS displayed a significantly greater turnover of DA to HVA compared to their saline controls (Fig 4G t = 4.940, df = 7, *p*<0.01). However, no difference in the ratio of DA to HVA between MPTP-treated animals receiving either PBS or activin A was found (t = 2.453, df = 5, *p* = 0.0578).

We also investigated if activin A was able to maintain its increase in striatal NE levels at 8 weeks after the administration of MPTP. As expected, two-way ANOVA demonstrated no significant overall effect of MPTP (F_(1,13)_ = 0.7078 *p* = 0.4154, n = 3-5/group) on striatal NE levels. However in contrast to our previous results we found no significant overall effect of drug treatment (F_(1,13)_ = 0.3297 *p* = 0.5756), suggesting activin A does not increase NE levels in the striatum 8 weeks after lesioning with MPTP (Fig 4H).

### Activin A does not protect dopamine transporter levels 8 weeks after MPTP

To further investigate the potential long term effects of activin A, we quantified DAT expression in the striatum 8 weeks after MPTP administration. While two-way ANOVA revealed a significant overall effect of MPTP on DAT expression (F_(1,42)_ = 26.80 *p*<0.001, n = 10-13/group), no significant effect of activin A was found (F_(1,42)_ = 0.08766 *p* = 0.7686). *Post hoc* analysis demonstrated that MPTP resulted in a significant decrease in DAT levels in animals receiving PBS (*p*<0.001), indicating that degeneration in the striatum is maintained up to 8 weeks (Fig 4I and 4J). However, while activin A administration did not alter baseline levels of DAT expression (*p*>0.9999), animals receiving activin A had significantly decreased DAT expression following MPTP lesioning at 8 weeks compared to saline controls (*p*<0.01) suggesting that activin A is unable to prevent long-term degeneration in the striatum following MPTP.

### Activin A decreases astrocyte and microglial numbers following MPTP

Numerous studies have shown that in addition to its selective degeneration of the dopaminergic system, the MPTP model of PD results in a significant increase in inflammatory cells in the SNpc [7,33–35]. It has been previously demonstrated that activin A administration results in a decrease in the total number of astrocytes and microglia in the hippocampus following an excitotoxic injury, thus demonstrating a potential anti-inflammatory effect of activin A [16]. We therefore investigated if activin A displayed similar anti-inflammatory properties following MPTP administration by quantifying the number of astrocytes and microglia via stereological analysis of GFAP and Iba1 immunoreactive cells, respectively, in the SNpc. Two-way ANOVA demonstrated a significant overall effect of MPTP on both GFAP (F_(1,10)_ = 19.41 p<0.01, n = 3-4/group) and Iba1 (F_(1,11)_ = 41.36 *p*<0.001, n = 3-4/group) cell numbers in the SNpc. Interestingly, there was no significant overall effect of drug treatment on GFAP positive cells (F_(1,10)_ = 4.484 *p* = 0.0603), however, a significant overall effect of drug treatment on Iba1 positive cells (F_(1,11)_ = 6.232 *p*<0.05) was found.

**Fig 5:**
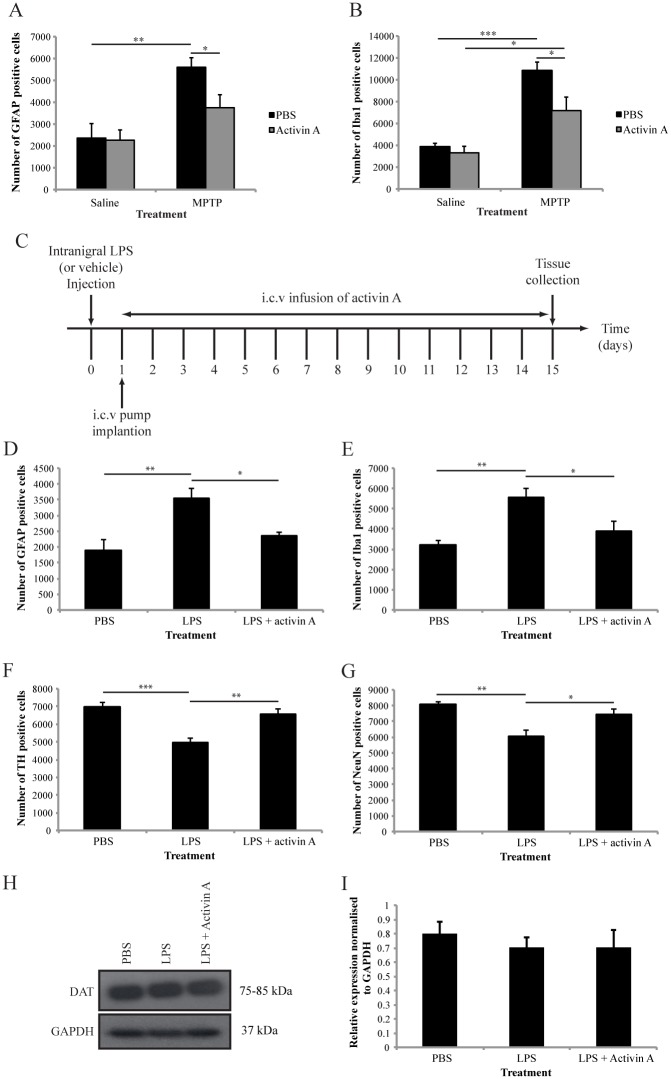
Activin A decreases MPTP and LPS-induced inflammation. Stereological quantification of the left SNpc demonstrated activin A significantly decreased the number of GFAP-immunoreactive cells (A) and Iba1-immunoreactive cells (B) in the SNpc following MPTP. (C) Timeline detailing LPS experimental procedures. Stereological quantification demonstrated activin A significantly reduces the LPS-induced increase in GFAP (D) and Iba1 (E) positive cells and subsequent loss of TH (F) and NeuN (G) positive cells. (H) Representative images of DAT and GAPDH expression. (I) Western blot analysis of striatal protein extracts revealed LPS does not alter DAT expression. All values represent the mean ± SEM. *p<0.05, **p<0.01, ***p<0.001. N = 3-5/group.

*Post hoc* analysis demonstrated no significant difference in GFAP positive cells between animals receiving PBS or activin A in the saline treated groups (*p*>0.9999), indicating that activin A does not alter the baseline level of astrocyte populations (Fig 5A). As expected, MPTP administration significantly increased GFAP positive cells in animals receiving PBS, compared to saline controls (*p*<0.01) indicating that MPTP was able to induce an inflammatory response (Fig 5A). We also found that activin A significantly lowered the number of GFAP positive cells following MPTP administration compared to controls (*p*<0.05) to levels that were not statistically significant different compared to animals receiving activin A and saline (*p* = 0.1332) suggesting that activin A was able to completely dampen the MPTP-induced astrocytic response.

Similarly, no significant difference in Iba1 positive cells was found between animals receiving PBS or activin A in the saline treated groups (*p*>0.9999), indicating that activin A does not alter the baseline level of microglial populations. As expected, MPTP significantly increased Iba1 positive cells in animals receiving PBS, compared to saline controls (*p*<0.001 Fig 5B). However, MPTP administration also increased the number of Iba1 positive cells in animals receiving activin A (*p*<0.05). Regardless, the increase in Iba1 positive cells was smaller in animals receiving activin A (Fig 5B), with a significant difference between animals receiving PBS and activin A in the MPTP treated groups (*p*<0.05). Together, these results indicate a potential anti-inflammatory effect of activin A, with the growth factor able to reduce both astrocyte and microglia populations following MPTP.

### Activin A decreases LPS-induced inflammation

LPS is a well-known potent inducer of inflammation, resulting in the production of pro-inflammatory cytokines and the subsequent activation of both astrocytes and microglia. To investigate if activin A is neuroprotective against a direct inflammatory mechanism we administered LPS (or vehicle PBS) directly into the SN, with or without activin A, and quantified the remaining number of astrocytes and microglial numbers via stereology (Fig 5C).

As expected, one-way ANOVA demonstrated a significant difference of both GFAP (F_(2,10)_ = 16.51, df = 2, *p*<0.001, n = 4-5/group) and Iba1 (F_(2,12)_ = 8.348 *p*<0.01, n = 4-5/group) positive cells numbers in the SNpc between treatment groups. *Post hoc* analysis revealed a significant difference between animals injected with PBS and animals injected with LPS alone for both GFAP (*p*<0.001) and Iba1 positive cells (*p*<0.01), indicating that LPS is able to induce a significant inflammatory response in the SNpc (Fig 5D and 5E). Furthermore, animals treated with activin A displayed fewer GFAP (*p*<0.5) and Iba1 (*p*<0.05) positive cells compared to animals injected with LPS alone. Interestingly, there was no significant difference in either GFAP positive cells (*p* = 0.1854) or Iba1 positive cells (*p* = 0.8269) between animals receiving activin A and those injected with PBS. Together, these results demonstrate that activin A administration significantly decreases LPS-induced increases in inflammatory cell populations in the SNpc.

### Activin A protects against LPS-induced cell death in the SNpc

To investigate if LPS-induced inflammation results in cell death in the SNpc and if activin A is able to protect against this degeneration, we quantified the number of TH and NeuN positive cells via stereology. One-way ANOVA demonstrated a significant difference of both TH positive (F_(2,12)_ = 15.02 *p*<0.001, n = 5/group) and NeuN positive (F_(2,12)_ = 12.26 *p*<0.01, n = 5/group) cell numbers in the SNpc between treatment groups. *Post hoc* analysis revealed a significant difference between animals injected with PBS and animals injected with LPS alone for both TH (Fig 5F; *p*<0.001) and NeuN positive cells (Fig 5G; *p*<0.01). However SN injection of LPS was unable to induce changes to DAT expression across all treatment groups (F_(2,12)_ = 0.3326, *p* = 0.7303), indicating that LPS is able to induce cell death in the SNpc but not degeneration in the striatum (Fig 5H and 5I). Furthermore animals treated with activin A displayed more TH positive (*p*<0.01) and NeuN positive (*p*<0.05) cells in the SNpc compared to animals injected with LPS alone. Most excitingly, there was no significant difference in either TH positive cells (*p* = 0.8708) or NeuN positive cells (*p* = 0.4610) between animals receiving activin A and those injected with PBS (Fig 5F–5G). Together, these results demonstrate that activin A is able to completely protect against cell death in the SN via an anti-inflammatory mechanism.

## Discussion

The use of growth factors has received intense focus in recent years as a potential therapy to halt or even reverse the progressive DA neuronal death in PD, based on their ability to promote induction, specification, survival and maturation of developing neurons within the CNS. Following promising neuroprotective and neurorestorative results in animal models, clinical trials of the growth factors GDNF and Neurturin in PD patients were conducted. However, the results of these clinical trials proved largely disappointing, with some endpoints not met, site delivery and retrograde transport issues, and the presence of unwanted lesions dampening results [36–40]. Despite these translational issues, optimism remains that growth factors, and in particular those of the TGF-β superfamily, will prove useful as a therapeutic intervention for PD. However, the mechanisms by which these growth factors ameliorate PD pathology still remain to be fully understood.

While the neuroprotective potential of activin A has been demonstrated previously in the hippocampus following acute brain injury [3], the first evidence that activin A may also exert neuroprotective effects in PD-related CNS regions was revealed when administration of the growth factor significantly attenuated degeneration induced by 6-OHDA [5] and MPP^+^ [4] *in vitro*. This study is the first to demonstrate that the anti-inflammatory role of activin A may contribute to its neuroprotective effects in an *in vivo* model of PD.

The MPTP model of PD remains the most widely used model to study potential neuroprotective therapeutic targets as it replicates the selective death of dopaminergic neurons within the SN. It has been previously reported that an acute regimen of MPTP results in a loss of dopaminergic cell bodies in the SN that is stable by 7 days after MPTP administration [41]. Following infusion of activin A (or vehicle) for 1 week stereological analysis revealed that administration of activin A for 7 days resulted in a significant protection of both dopaminergic and total neuron populations in the SNpc against MPTP-induced toxicity, suggesting that administration of activin A throughout the entire period of MPTP toxicity offers profound neuroprotection within the SN. It would be interesting for future translational studies to determine if these surviving neurons exhibited the same electrophysiological properties as those found in unlesioned animals.

The loss of DA producing cells within the SN of PD patients results in a subsequent loss of DA within the striatum, and ultimately a disruption of the finely tuned signaling of the BG. As we demonstrated that activin A resulted in significant protection of dopaminergic and total neuron populations in the SN, we hypothesized that exogenous activin A would also result in a subsequent protection of DA levels. However, activin A treatment was unable to attenuate this loss of DA levels, or its metabolites DOPAC and HVA. Furthermore this was not due to activin A altering the catabolism of DA, with no difference in the ratio of DA to HVA found between animals receiving vehicle or activin A. These results suggest that while activin A protects against MPTP-induced degeneration of nigral cell bodies, this neuroprotection does not translate to a subsequent protection of DA levels in the striatum. Interestingly, this finding replicates a previous study in which cyclooxygenase-2 (COX-2) deficient mice exhibited reduced dopaminergic cell loss following MPTP, but maintained a deficit of approximately 70% striatal DA levels [42].

Standard MPTP dosing regimes, including the acute MPTP protocol used in this study, result in profound striatal DA depletion with little to no evidence of alterations of NE content in mice [43–45]. It is therefore interesting to note that exogenous activin A significantly increases levels of striatal NE in both saline and MPTP injected animals. In nerve terminals containing dopamine-β-hydroxylase, NE is formed in the next step in the catecholamine synthesis pathway beyond DA production, and has been demonstrated to be greatly reduced in several brain regions in PD patients [46]. Furthermore, it has been suggested that activation of α_2_ adrenergic receptors, thus decreasing NE neurotransmission, can facilitate movements produced by the activation of the direct pathway of the basal ganglia, thus highlighting enhanced α_2_ receptor stimulation as a potential mechanism underlying L-dopa-induced dyskinesias [47]. Indeed, α_2_ adrenergic antagonists have been shown to reduce L-dopa-induced motor effects in 6-OHDA lesioned rodents [48,49]. Therefore the role of activin A in NE transmission, and subsequent effect on motor function would warrant further investigations.

The production of DA within the CNS is a two-step biosynthesis that takes place within the cytosol of catecholaminergic neurons, beginning with the hydroxylation of L-tyrosine by TH to yield DOPA [50]. TH activation by phosphorylation is the primary mechanism responsible for the maintenance of catecholamine levels after catecholamine secretion in tissues and occurs at serine 19, 31 and 40 by various kinases to increase stability and/or activity of TH [26,50]. To investigate if the failure of activin A to restore striatal DA levels resulted from alterations to DA synthesis, we quantified changes in striatal expression of phosphorylation at the two major sites responsible for TH activity, ser40 and ser31. We found that following MPTP administration, there was a significant increase in phosphorylation at ser40 and a concomitant decrease at ser31 in animals that were infused with activin A. These results suggest that in the presence of nigrostriatal degeneration and DA loss, a compensatory mechanism involving activin A to increase the phosphorylation of TH at ser40, the most important site in the regulation of TH activity, occurs in an attempt to stimulate DA production and replenish the DA that is lost. However, despite this increase in phosphorylation at ser40, this does not result in an increase in striatal DA levels, suggesting that activin A may (1) alter the DA production pathway further downstream or (2) inhibit the activity of protein phosphatase 2A (PP2A), thus inhibiting the dephosphorylation of TH at ser40. Further experiments investigating the effects of activin A on other important regulators of DA biosynthesis such as aromatic amino acid decarboxylase (AADC), as well as its potential to inhibit PP2A would be required to understand activin A’s role in regulating DA levels in the striatum more fully.

In the striatum, the dopamine transporter plays an important role for maintaining sufficient DA levels for release into the synaptic cleft and is the primary determinant of the lifetime of extracellular DA, thus when striatal DAT loss reaches levels equivalent to those seen upon presentation of locomotor symptoms, a concomitant deficit in DA is produced [27–29]. It is therefore possible that the decrease in striatal DA levels in animals receiving activin A, despite the increase in survival of DA producing cells and an increase in TH phosphorylation, is due to the degeneration of the DAT in the projecting fibers from the nigral cell bodies. Quantification of DAT expression in striatal protein homogenates demonstrated no changes between vehicle and activin A treated animals following MPTP. While it could be suggested this may be due to an inability of i.c.v infusion to allow for insufficient levels of activin to reach the striatal region, our quantification of activin A in this region demonstrates this is not a factor. However despite a trend towards loss of DAT expression 1 week after MPTP, this effect was not significant in either treatment group, suggesting that this timeframe was insufficient for loss of striatal integrity.

There is substantial evidence that spontaneous recovery is able to occur in rodents that have been rendered parkinsonian by MPTP, similar to the compensatory effects that occur in the early stages of PD [51]. We therefore investigated if the neuroprotective effects of activin A extended past the initial degenerative phase by examining levels of nigrostriatal protection 8 weeks after the administration of MPTP. We found that activin A treatment was able to maintain protection of both dopaminergic and total neuronal populations in the SNpc in the long-term, even in the absence of continued activin A infusion. This result is similar to that seen with previous studies, in which short-term infusion of activin A regulated neurogenesis in the hippocampus up to 5 weeks later [16]. However, much like that seen in the earlier experiments of this study, activin A was not able to subsequently restore levels of striatal DA, or its metabolites DOPAC and HVA. A study conducted by Jones et al., (1998) suggests that the maintenance of normal stores of DA are dependent on recycled rather than newly synthesized DA [29], therefore the loss of striatal DAT expression in activin A treated animals 8 weeks after MPTP, allowing for limited extracellular DA to be taken up into presynaptic terminals rather than be degraded, may not be enough to maintain striatal DA levels. In contrast to our previous results, NE levels were unchanged in activin A infused animals, demonstrating that the increase in NE levels seen 8 days after MPTP administration is not maintained long term. This suggests that activin A may need to be constantly administrated to the brain to maintain consistent levels of NE in the striatum.

Our findings here, showing a neuroprotective effect that appears to be localized to the SN, reflects a similar outcome to that of a recently published study in which activin A significantly protected nigral neurons but not striatal DA and DAT levels against 6-OHDA-induced toxicity *in vivo* [6]. Combined, this raises a number of interesting points. It is possible that proximity of administration of activin A to the site of degeneration is required for any neuroprotective effects to occur in the striatum, much like that seen with GDNF and Neurturin in animal models of PD [52–57]. While our method of i.c.v infusion resulted in diffusion to the striatum, it may be that there is still insufficient activin A levels to inhibit striatal loss. However, it is unknown, at least in the MPTP model where degeneration is not localized to one area, if any neuroprotection as a result of administration of activin A in the striatal region would come at a consequence of neuroprotection in the SN.

While it has long been known that growth factors support and promote the survival of midbrain neurons in animal models of PD, the exact mechanisms of this protection remains to be fully elucidated. It has previously been demonstrated that i.c.v administration of activin A exerts profound anti-inflammatory effects in the hippocampus following an acute excitotoxic injury through decreased total astrocyte and microglial numbers and inhibition of pro-inflammatory cytokine release [16], suggesting a potential mechanism for its neuroprotective effects. Systemic injections of MPTP result in rapid astroglia and microglia-mediated responses such as increases in cell numbers and changes in morphology, including larger cell bodies and thickening of processes [58,59], making this model ideal to investigate activin A-mediated inflammatory changes. Indeed, activin A suppressed the MPTP-induced inflammatory response, with stereological quantification demonstrating significantly fewer number of astrocytes and microglial cells in the SNpc in activin A infused animals. Given that previous studies have shown that in response to the neurotoxic damage induced by MPTP in mice, neurotrophic factors such as GDNF are upregulated by glia to serve a neuroprotective function [60,61], it is therefore not unexpected that the neuroprotective and anti-inflammatory action of activin A may linked.

However, while showing a significant reduction in inflammatory cell populations, these results do not demonstrate that activin A’s neuroprotective effects against MPTP-induced toxicity are directly due to its anti-inflammatory properties. We therefore investigated the potential of activin A to inhibit a direct stimulation of inflammation by injection of LPS into the substantia nigra, which has been previously demonstrated to result in significant degeneration of dopaminergic neurons [62]. When administered after the inflammatory process had already begun, activin A significantly decreased the number of astrocytes and microglia 2 weeks after the injection of LPS. These results confirm previous studies in which activin A inhibits the function of LPS-activated macrophages *in vitro* and *in vivo* [63–65]. Furthermore, while LPS resulted in a significant degeneration within the SN but not in the striatum, activin A administration resulted in the complete protection of dopaminergic and total neuron populations. A study conducted by Li et al (2013) showed that activin A inhibited LPS-induced changes via down-regulating TLR4 not TLR2 [66], suggesting a potential avenue for exploring the mechanisms underlying activin A-mediated inflammation. These results indicate that much like its effects in the hippocampus, activin A is a potent anti-inflammatory agent in the midbrain, a region with the highest density of microglia [67], and it is this anti-inflammatory property that contributes to its neuroprotective effects, a previously unknown action of activin A. Investigations into the effect of activin A on other regulators of inflammation following MPTP toxicity such as cytokine release, nitric oxide levels, and quantification of activated glial cells via morphology changes will further consolidate this relationship.

## Conclusions

Despite decades of research, L-Dopa remains the single most effective treatment for PD, however this treatment strategy comes with its own set of drawbacks and furthermore does not address the underlying degeneration that is characteristic of the disease. Growth factors have demonstrated significant neuroprotective effects in multiple animal models of PD, however the mechanism by which they provide these effects has yet to be fully understood. The findings presented in this study provide the first evidence that exogenous activin A is able to significantly increase the survival of midbrain dopaminergic and total neuron populations through a potential anti-inflammatory mechanism.

## Supporting Information

S1 Fig Activin A levels in the midbrain and striatum. ELISA analysis demonstrates that i.c.v administration of activin A significantly increased levels of activin A in both the midbrain (A) and striatum (B) when analysed 24 hours after lesioning with MPTP. All values represent the mean ± SEM. ***p<0.001. N = 9-13/group.
(TIF)

S1 Dataset Raw data used for Figs 1–5 and S1 Fig. N/A = result not available due to insufficient tissue.
(XLSX)

## References

[1] Hughes PE, Alexi T, Walton M, Williams CE, Dragunow M, Clark RG, et al (1999) Activity and injury-dependent expression of inducible transcription factors, growth factors and apoptosis-related genes within the central nervous system. Prog Neurobiol 57: 421–450. 10080384 10.1016/s0301-0082(98)00057-4

[2] Kordower JH, Bjorklund A (2013) Trophic factor gene therapy for Parkinson's disease. Mov Disord 28: 96–109. 10.1002/mds.25344 23390096

[3] Tretter YP, Hertel M, Munz B, ten Bruggencate G, Werner S, Alzheimer C (2000) Induction of activin A is essential for the neuroprotective action of basic fibroblast growth factor in vivo. Nat Med 6: 812–815. 10.1038/77548 10888932

[4] Krieglstein K, Suter-Crazzolara C, Fischer WH, Unsicker K (1995) TGF-beta superfamily members promote survival of midbrain dopaminergic neurons and protect them against MPP+ toxicity. EMBO J 14: 736–742. 7882977 10.1002/j.1460-2075.1995.tb07052.x PMC398139

[5] Kupershmidt L, Amit T, Bar-Am O, Youdim MB, Blumenfeld Z (2007) The neuroprotective effect of Activin A and B: implication for neurodegenerative diseases. J Neurochem 103: 962–971. 10.1111/j.1471-4159.2007.04785.x 17680997

[6] Stayte S, Rentsch P, Li KM, Vissel B (2015) Activin A protects midbrain neurons in the 6-hydroxydopamine mouse model of Parkinson's disease. PLoS One 10: e0124325 10.1371/journal.pone.0124325 25902062 PMC4406584

[7] Barcia C, Ros CM, Annese V, Gomez A, Ros-Bernal F, Aguado-Yera D, et al (2011) IFN-gamma signaling, with the synergistic contribution of TNF-alpha, mediates cell specific microglial and astroglial activation in experimental models of Parkinson's disease. Cell Death Dis 2: e142 10.1038/cddis.2011.17 21472005 PMC3122054

[8] Barkholt P, Sanchez-Guajardo V, Kirik D, Romero-Ramos M (2012) Long-term polarization of microglia upon alpha-synuclein overexpression in nonhuman primates. Neuroscience 208: 85–96. 10.1016/j.neuroscience.2012.02.004 22342967

[9] Gao HM, Hong JS (2008) Why neurodegenerative diseases are progressive: uncontrolled inflammation drives disease progression. Trends Immunol 29: 357–365. 10.1016/j.it.2008.05.002 18599350 PMC4794280

[10] Sanchez-Guajardo V, Febbraro F, Kirik D, Romero-Ramos M (2010) Microglia acquire distinct activation profiles depending on the degree of alpha-synuclein neuropathology in a rAAV based model of Parkinson's disease. PLoS One 5: e8784 10.1371/journal.pone.0008784 20098715 PMC2808388

[11] Wachter B, Schurger S, Rolinger J, von Ameln-Mayerhofer A, Berg D, Wagner HJ, et al (2011) Effect of 6-hydroxydopamine (6-OHDA) on proliferation of glial cells in the rat cortex and striatum: evidence for de-differentiation of resident astrocytes. Cell Tissue Res 342: 147–160. 10.1007/s00441-010-1061-x 20976472

[12] Appel E, Kolman O, Kazimirsky G, Blumberg PM, Brodie C (1997) Regulation of GDNF expression in cultured astrocytes by inflammatory stimuli. Neuroreport 8: 3309–3312. 9351662 10.1097/00001756-199710200-00023

[13] Hoban DB, Howard L, Dowd E (2015) GDNF-secreting mesenchymal stem cells provide localized neuroprotection in an inflammation-driven rat model of Parkinson's disease. Neuroscience 303: 402–411. 10.1016/j.neuroscience.2015.07.014 26166730

[14] Mogi M, Togari A, Kondo T, Mizuno Y, Komure O, Kuno S, et al (1999) Brain-derived growth factor and nerve growth factor concentrations are decreased in the substantia nigra in Parkinson's disease. Neurosci Lett 270: 45–48. 10454142 10.1016/s0304-3940(99)00463-2

[15] Suh HS, Zhao ML, Derico L, Choi N, Lee SC (2013) Insulin-like growth factor 1 and 2 (IGF1, IGF2) expression in human microglia: differential regulation by inflammatory mediators. J Neuroinflammation 10: 37 10.1186/1742-2094-10-37 23497056 PMC3607995

[16] Abdipranoto-Cowley A, Park JS, Croucher D, Daniel J, Henshall S, Galbraith S, et al (2009) Activin A is essential for neurogenesis following neurodegeneration. Stem Cells 27: 1330–1346. 10.1002/stem.80 19489097 PMC2733378

[17] Gundersen HJ, Jensen EB (1987) The efficiency of systematic sampling in stereology and its prediction. J Microsc 147: 229–263. 3430576 10.1111/j.1365-2818.1987.tb02837.x

[18] Paxinos G, Franklin K (2001) The mouse brain in stereotaxic coordinates. San Diego: Academic Press.

[19] Kastner A, Herrero MT, Hirsch EC, Guillen J, Luquin MR, Javoy-Agid F, et al (1994) Decreased tyrosine hydroxylase content in the dopaminergic neurons of MPTP-intoxicated monkeys: effect of levodopa and GM1 ganglioside therapy. Ann Neurol 36: 206–214. 10.1002/ana.410360213 7914399

[20] Kozina EA, Khakimova GR, Khaindrava VG, Kucheryanu VG, Vorobyeva NE, Krasnov AN, et al (2014) Tyrosine hydroxylase expression and activity in nigrostriatal dopaminergic neurons of MPTP-treated mice at the presymptomatic and symptomatic stages of parkinsonism. J Neurol Sci 340: 198–207. 10.1016/j.jns.2014.03.028 24768159

[21] Xu Z, Cawthon D, McCastlain KA, Slikker W Jr., Ali SF (2005) Selective alterations of gene expression in mice induced by MPTP. Synapse 55: 45–51. 10.1002/syn.20089 15499605

[22] Beaulieu JM, Gainetdinov RR (2011) The physiology, signaling, and pharmacology of dopamine receptors. Pharmacol Rev 63: 182–217. 10.1124/pr.110.002642 21303898

[23] Vaughan RA, Foster JD (2013) Mechanisms of dopamine transporter regulation in normal and disease states. Trends Pharmacol Sci 34: 489–496. 10.1016/j.tips.2013.07.005 23968642 PMC3831354

[24] Gesi M, Soldani P, Giorgi FS, Santinami A, Bonaccorsi I, Fornai F (2000) The role of the locus coeruleus in the development of Parkinson's disease. Neurosci Biobehav Rev 24: 655–668. 10940440 10.1016/s0149-7634(00)00028-2

[25] Rommelfanger KS, Weinshenker D (2007) Norepinephrine: The redheaded stepchild of Parkinson's disease. Biochem Pharmacol 74: 177–190. 10.1016/j.bcp.2007.01.036 17416354

[26] Dunkley PR, Bobrovskaya L, Graham ME, von Nagy-Felsobuki EI, Dickson PW (2004) Tyrosine hydroxylase phosphorylation: regulation and consequences. J Neurochem 91: 1025–1043. 10.1111/j.1471-4159.2004.02797.x 15569247

[27] Bezard E, Dovero S, Prunier C, Ravenscroft P, Chalon S, Guilloteau D, et al (2001) Relationship between the appearance of symptoms and the level of nigrostriatal degeneration in a progressive 1-methyl-4-phenyl-1,2,3,6-tetrahydropyridine-lesioned macaque model of Parkinson's disease. J Neurosci 21: 6853–6861. 11517273 10.1523/JNEUROSCI.21-17-06853.2001 PMC6763089

[28] Jaber M, Dumartin B, Sagne C, Haycock JW, Roubert C, Giros B, et al (1999) Differential regulation of tyrosine hydroxylase in the basal ganglia of mice lacking the dopamine transporter. Eur J Neurosci 11: 3499–3511. 10564358 10.1046/j.1460-9568.1999.00764.x

[29] Jones SR, Gainetdinov RR, Jaber M, Giros B, Wightman RM, Caron MG (1998) Profound neuronal plasticity in response to inactivation of the dopamine transporter. Proc Natl Acad Sci U S A 95: 4029–4034. 9520487 10.1073/pnas.95.7.4029 PMC19957

[30] Hallman H, Olson L, Jonsson G (1984) Neurotoxicity of the meperidine analogue N-methyl-4-phenyl-1,2,3,6-tetrahydropyridine on brain catecholamine neurons in the mouse. Eur J Pharmacol 97: 133–136. 6607840 10.1016/0014-2999(84)90521-1

[31] Heikkila RE, Sonsalla PK (1992) The MPTP-treated mouse as a model of parkinsonism: how good is it? Neurochem Int 20 Suppl: 299S–303S. 1365446 10.1016/0197-0186(92)90256-q

[32] Willis GL, Donnan GA (1987) Histochemical, biochemical and behavioural consequences of MPTP treatment in C-57 black mice. Brain Res 402: 269–274. 3493826 10.1016/0006-8993(87)90033-3

[33] Grunblatt E, Mandel S, Maor G, Youdim MB (2001) Gene expression analysis in N-methyl-4-phenyl-1,2,3,6-tetrahydropyridine mice model of Parkinson's disease using cDNA microarray: effect of R-apomorphine. J Neurochem 78: 1–12. 10.1046/j.1471-4159.2001.00397.x 11432968

[34] McGeer PL, Schwab C, Parent A, Doudet D (2003) Presence of reactive microglia in monkey substantia nigra years after 1-methyl-4-phenyl-1,2,3,6-tetrahydropyridine administration. Ann Neurol 54: 599–604. 10.1002/ana.10728 14595649

[35] Yasuda Y, Shimoda T, Uno K, Tateishi N, Furuya S, Yagi K, et al (2008) The effects of MPTP on the activation of microglia/astrocytes and cytokine/chemokine levels in different mice strains. J Neuroimmunol 204: 43–51. 10.1016/j.jneuroim.2008.08.003 18817984

[36] AMGEN (2005) Following Complete Review of Phase 2 Trial Data Amgen Confirms Decision to Halt GDNF Study; Comprehensive Review of Scientific Findings, Patient Safety, Drove Decision. Thousand Oaks: AMGEN.

[37] Bartus RT, Baumann TL, Siffert J, Herzog CD, Alterman R, Boulis N, et al (2013) Safety/feasibility of targeting the substantia nigra with AAV2-neurturin in Parkinson patients. Neurology 80: 1698–1701. 10.1212/WNL.0b013e3182904faa 23576625 PMC3716474

[38] Bartus RT, Herzog CD, Chu Y, Wilson A, Brown L, Siffert J, et al (2011) Bioactivity of AAV2-neurturin gene therapy (CERE-120): differences between Parkinson's disease and nonhuman primate brains. Mov Disord 26: 27–36. 10.1002/mds.23442 21322017 PMC6333467

[39] Ceregene (2013) Ceregene reports data from Parkinson's disease Phase 2b study. San Diego.

[40] Kordower JH, Palfi S, Chen EY, Ma SY, Sendera T, Cochran EJ, et al (1999) Clinicopathological findings following intraventricular glial-derived neurotrophic factor treatment in a patient with Parkinson's disease. Ann Neurol 46: 419–424. 10482276 10.1002/1531-8249(199909)46:3<419::aid-ana21>3.0.co;2-q

[41] Jackson-Lewis V, Jakowec M, Burke RE, Przedborski S (1995) Time-Course and Morphology of Dopaminergic Neuronal Death Caused by the Neurotoxin 1-Methyl-4-Phenyl-1,2,3,6-Tetrahydropyridine. Neurodegeneration 4: 257–269. 8581558 10.1016/1055-8330(95)90015-2

[42] Feng ZH, Wang TG, Li DD, Fung P, Wilson BC, Liu B, et al (2002) Cyclooxygenase-2-deficient mice are resistant to 1-methyl-4-phenyl1, 2, 3, 6-tetrahydropyridine-induced damage of dopaminergic neurons in the substantia nigra. Neurosci Lett 329: 354–358. 12183047 10.1016/s0304-3940(02)00704-8

[43] Archer T, Fredriksson A (2006) Influence of noradrenaline denervation on MPTP-induced deficits in mice. J Neural Transm 113: 1119–1129. 10.1007/s00702-005-0402-5 16362627

[44] Fornai F, Alessandri MG, Torracca MT, Bassi L, Corsini GU (1997) Effects of noradrenergic lesions on MPTP/MPP+ kinetics and MPTP-induced nigrostriatal dopamine depletions. J Pharmacol Exp Ther 283: 100–107. 9336313

[45] German DC, Liang CL, Manaye KF, Lane K, Sonsalla PK (2000) Pharmacological inactivation of the vesicular monoamine transporter can enhance 1-methyl-4-phenyl-1,2,3,6-tetrahydropyridine-induced neurodegeneration of midbrain dopaminergic neurons, but not locus coeruleus noradrenergic neurons. Neuroscience 101: 1063–1069. 11113355 10.1016/s0306-4522(00)00385-7

[46] Agid Y, Javoy-Agid F, Ruberg M (1987) Biochemistry of neurotransmitters in Parkinson's disease In: Marsden C, Fahn S, editors. Movement Disorders 2. New York: Butterworths pp. 166–230.

[47] Hill MP, Brotchie JM (1999) The adrenergic receptor agonist, clonidine, potentiates the anti-parkinsonian action of the selective kappa-opioid receptor agonist, enadoline, in the monoamine-depleted rat. Br J Pharmacol 128: 1577–1585. 10.1038/sj.bjp.0702943 10602339 PMC1571785

[48] Barnum CJ, Bhide N, Lindenbach D, Surrena MA, Goldenberg AA, Tignor S, et al (2012) Effects of noradrenergic denervation on L-DOPA-induced dyskinesia and its treatment by alpha- and beta-adrenergic receptor antagonists in hemiparkinsonian rats. Pharmacol Biochem Behav 100: 607–615. 10.1016/j.pbb.2011.09.009 21978941 PMC3242909

[49] Henry B, Crossman AR, Brotchie JM (1998) Characterization of enhanced behavioral responses to L-DOPA following repeated administration in the 6-hydroxydopamine-lesioned rat model of Parkinson's disease. Exp Neurol 151: 334–342. 10.1006/exnr.1998.6819 9628768

[50] Meiser J, Weindl D, Hiller K (2013) Complexity of dopamine metabolism. Cell Commun Signal 11: 34 10.1186/1478-811X-11-34 23683503 PMC3693914

[51] Sedelis M, Schwarting RK, Huston JP (2001) Behavioral phenotyping of the MPTP mouse model of Parkinson's disease. Behav Brain Res 125: 109–125. 11682102 10.1016/s0166-4328(01)00309-6

[52] Eberling JL, Kells AP, Pivirotto P, Beyer J, Bringas J, Federoff HJ, et al (2009) Functional effects of AAV2-GDNF on the dopaminergic nigrostriatal pathway in parkinsonian rhesus monkeys. Hum Gene Ther 20: 511–518. 10.1089/hum.2008.201 19254173 PMC2725183

[53] Eslamboli A, Cummings RM, Ridley RM, Baker HF, Muzyczka N, Burger C, et al (2003) Recombinant adeno-associated viral vector (rAAV) delivery of GDNF provides protection against 6-OHDA lesion in the common marmoset monkey (Callithrix jacchus). Exp Neurol 184: 536–548. 14637123 10.1016/j.expneurol.2003.08.007

[54] Herzog CD, Brown L, Kruegel BR, Wilson A, Tansey MG, Gage FH, et al (2013) Enhanced neurotrophic distribution, cell signaling and neuroprotection following substantia nigral versus striatal delivery of AAV2-NRTN (CERE-120). Neurobiol Dis 58: 38–48. 10.1016/j.nbd.2013.04.011 23631873

[55] Johnston LC, Eberling J, Pivirotto P, Hadaczek P, Federoff HJ, Forsayeth J, et al (2009) Clinically relevant effects of convection-enhanced delivery of AAV2-GDNF on the dopaminergic nigrostriatal pathway in aged rhesus monkeys. Hum Gene Ther 20: 497–510. 10.1089/hum.2008.137 19203243 PMC2767387

[56] Kirik D, Rosenblad C, Bjorklund A (2000) Preservation of a functional nigrostriatal dopamine pathway by GDNF in the intrastriatal 6-OHDA lesion model depends on the site of administration of the trophic factor. Eur J Neurosci 12: 3871–3882. 11069582 10.1046/j.1460-9568.2000.00274.x

[57] Wang L, Muramatsu S, Lu Y, Ikeguchi K, Fujimoto K, Okada T, et al (2002) Delayed delivery of AAV-GDNF prevents nigral neurodegeneration and promotes functional recovery in a rat model of Parkinson's disease. Gene Ther 9: 381–389. 10.1038/sj.gt.3301682 11960314

[58] Czlonkowska A, Kohutnicka M, Kurkowska-Jastrzebska I, Czlonkowski A (1996) Microglial reaction in MPTP (1-methyl-4-phenyl-1,2,3,6-tetrahydropyridine) induced Parkinson's disease mice model. Neurodegeneration 5: 137–143. 8819134 10.1006/neur.1996.0020

[59] Kohutnicka M, Lewandowska E, Kurkowska-Jastrzebska I, Czlonkowski A, Czlonkowska A (1998) Microglial and astrocytic involvement in a murine model of Parkinson's disease induced by 1-methyl-4-phenyl-1,2,3,6-tetrahydropyridine (MPTP). Immunopharmacology 39: 167–180. 9754903 10.1016/s0162-3109(98)00022-8

[60] Chen LW, Zhang JP, Kwok-Yan Shum D, Chan YS (2006) Localization of nerve growth factor, neurotrophin-3, and glial cell line-derived neurotrophic factor in nestin-expressing reactive astrocytes in the caudate-putamen of 1-methyl-4-phenyl-1,2,3,6-tetrahydropyridine-treated C57/Bl mice. J Comp Neurol 497: 898–909. 10.1002/cne.21014 16802332

[61] Chen PC, Vargas MR, Pani AK, Smeyne RJ, Johnson DA, Kan YW, et al (2009) Nrf2-mediated neuroprotection in the MPTP mouse model of Parkinson's disease: Critical role for the astrocyte. Proc Natl Acad Sci U S A 106: 2933–2938. 10.1073/pnas.0813361106 19196989 PMC2650368

[62] Castano A, Herrera AJ, Cano J, Machado A (1998) Lipopolysaccharide intranigral injection induces inflammatory reaction and damage in nigrostriatal dopaminergic system. J Neurochem 70: 1584–1592. 9580157 10.1046/j.1471-4159.1998.70041584.x

[63] Nusing RM, Mohr S, Ullrich V (1995) Activin A and retinoic acid synergize in cyclooxygenase-1 and thromboxane synthase induction during differentiation of J774.1 macrophages. Eur J Biochem 227: 130–136. 7851378 10.1111/j.1432-1033.1995.tb20368.x

[64] Vale W, Rivier J, Vaughan J, McClintock R, Corrigan A, Woo W, et al (1986) Purification and characterization of an FSH releasing protein from porcine ovarian follicular fluid. Nature 321: 776–779. 10.1038/321776a0 3012369

[65] Wang SY, Tai GX, Zhang PY, Mu DP, Zhang XJ, Liu ZH (2008) Inhibitory effect of activin A on activation of lipopolysaccharide-stimulated mouse macrophage RAW264.7 cells. Cytokine 42: 85–91. 10.1016/j.cyto.2008.01.010 18321725

[66] Li N, Cui X, Ge J, Li J, Niu L, Liu H, et al (2013) Activin A inhibits activities of lipopolysaccharide-activated macrophages via TLR4, not of TLR2. Biochem Biophys Res Commun 435: 222–228. 10.1016/j.bbrc.2013.04.077 23665022

[67] Yang TT, Lin C, Hsu CT, Wang TF, Ke FY, Kuo YM (2013) Differential distribution and activation of microglia in the brain of male C57BL/6J mice. Brain Struct Funct 218: 1051–1060. 10.1007/s00429-012-0446-x 22886465

