# Supplementary figures and images for: Activin A Inhibits MPTP and LPS-Induced Increases in Inflammatory Cell Populations and Loss of Dopamine Neurons in the Mouse Midbrain In Vivo
Source: PLoS One. 2017 Jan 25;12(1):e0167211. doi: 10.1371/journal.pone.0167211 (PMC5266209; doi:10.1371/journal.pone.0167211)

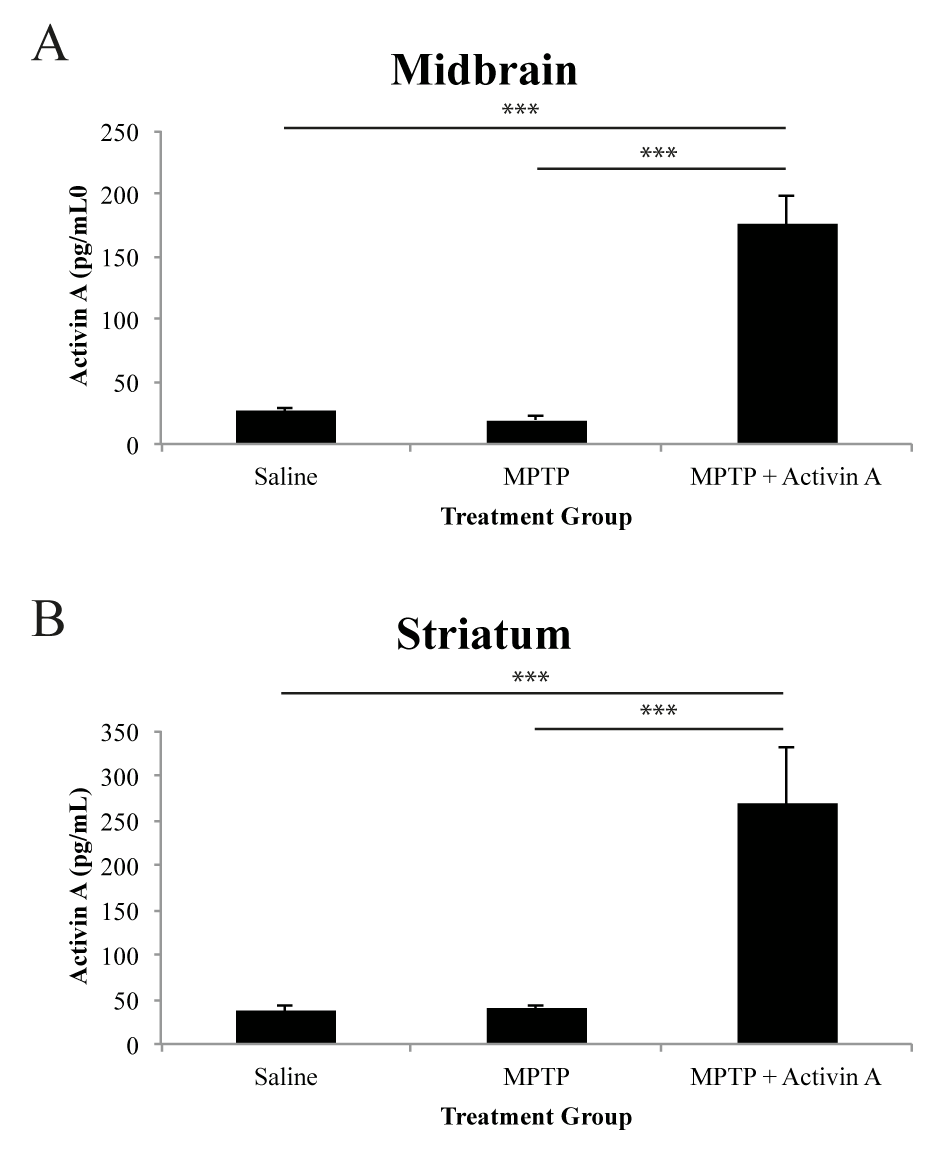

Supplement: S1 Fig — ELISA analysis demonstrates that i.c.v administration of activin A significantly increased levels of activin A in both the midbrain (A) and striatum (B) when analysed 24 hours after lesioning with MPTP. All values represent the mean ± SEM. ***p<0.001. N = 9-13/group. (TIF) [file pone.0167211.s001.tif]
